# ADULT-TO-PEDIATRIC LIVING DONOR LIVER TRANSPLANT IN RECIPIENTS >20 KG: A CASE SERIES OF FULL LEFT LOBE GRAFTS

**DOI:** 10.1590/0102-6720202400035e1828

**Published:** 2024-10-28

**Authors:** Catalina ORTIZ, José Donizeti MEIRA, Juan Carlos PATTILLO, Eduardo VIÑUELA, Nicholas JARUFE, Jorge MARTÍNEZ, Eduardo BRICEÑO, Martin DIB

**Affiliations:** 1 Pontificia Universidade Catolica do Chile, Department of Digestive Surgery, Hepato-Pancreato-Biliary Surgery Division – Santiago, Chile; 2 Universidade de São Paulo, Faculty of Medicine, Department of Gastroenterology, Digestive Surgery Division – São Paulo (SP), Brazil; 3 Pontificia Universidade Catolica do Chile, Pediatric Surgery Department – Santiago, Chile.

**Keywords:** Liver Transplantation, Living Donors, Liver Failure, Transplante de Fígado, Doadores Vivos, Insuficiência Hepática

## Abstract

**BACKGROUND:**

Chile presents one of the lowest organ donation rates, resulting in pediatric liver waitlist mortality rates up to 38.1%. Live donor liver transplantation is one of the main alternatives to decrease waitlist mortality, mostly utilized in our country for small children up to 20 kg.

**AIMS:**

The aim of this study was to report a three-case series of adult-to-pediatric living donor liver transplantation using a full left lobe graft.

**METHODS:**

We report three cases of children with more than 20 kg who received complete left hemi-grafts in different clinical scenarios. The indications and techniques adopted are discussed.

**RESULTS:**

Three children, two girls and one boy, aged 11, 7, and 3 years, were transplanted. The indications for transplant were fulminant hepatitis of autoimmune etiology, hepatoblastoma, and chronic liver failure due to autoimmune hepatitis, respectively. The evolution was satisfactory in all three children, and to date, all are well, approximately 12–24 months after the transplant.

**CONCLUSIONS:**

The use of a living donor left lateral segment (segments 2 and 3) has been successfully employed in pediatric liver transplantation. However, it is only suitable for infants and low-weight children. This approach using the whole left hemi-liver graft contributes to the reduction of small-for-size syndrome, mortality rate, and waiting times associated with deceased donors.

## RESUMO

**RACIONAL:**

O Chile apresenta uma das taxas de doação de órgãos mais baixa de todo o mundo, resultando em uma mortalidade pediátrica em fila de espera de até 38,1%. O transplante hepático com doador vivo é uma das principais alternativas para diminuir a mortalidade em fila de espera, geralmente utilizado para crianças pequenas, de até 20 kg.

**OBJETIVOS:**

Reportar uma série de três casos de transplante hepático com doador vivo utilizando enxertos de lobo esquerdo completo.

**MÉTODOS:**

São relatados três casos de crianças com mais de 20 kg que receberam hemi-enxertos esquerdos completos em diferentes cenários clínicos, todos eles com sucesso. São discutidas as indicações e técnicas adotadas.

**RESULTADOS:**

Foram transplantados, respectivamente, três crianças, duas femininas e um masculino, com 11, 7 e 3 anos. As indicações do transplante foram, respectivamente, hepatite fulminante de etiologia autoimune, hepatoblastoma e insuficiência hepática crônica por hepatite autoimune. A evolução foi satisfatória nas três crianças, sendo que até o momento, todos estão em bom estado geral, cerca de 12 a 24 meses após o transplante.

**CONCLUSÕES:**

O uso de um segmento lateral esquerdo de doador vivo (segmentos 2 e 3) tem sido empregado com sucesso em transplante de fígado pediátrico. No entanto, ele é adequado apenas para bebês e crianças de baixo peso. Essa abordagem utilizando o enxerto de todo o lobo esquerdo contribui para a redução de disfunção hepática pós transplante por volume reduzido, e menores taxas de mortalidade e tempos de espera associados a doadores falecidos.

## INTRODUCTION

In 2022, 465 solid organ transplants from deceased donors were performed in Chile, with a donation rate of 8.6 donors per million inhabitants^31^. Although the donation rate was higher than in the two previous years, it falls considerably short of the global maximum reported in Spain, which was 41.73 donors per million for the same year^9^. In this scenario, considering that the national mortality on the waiting list for pediatric liver transplantation due to chronic liver disease is 25.1%, rising to 38.1% in children under 2 years, it is crucial to enhance alternatives to reduce this gap, among which living donor liver transplantation plays a significant role^1,10^.

Traditionally, a left lateral graft has been used in pediatric recipients; however, in children of higher weight, it tends to be insufficient. The decision to implant a complete left graft should take into account the possibility of reconstructing the middle hepatic vein (MHV) in the recipient, which requires a detailed evaluation of the graft’s anatomy to ensure adequate outflow and prevent “small-for-size syndrome” (SFSS).

The objective of this report is to present a three-case series of adult-to-pediatric living donor liver transplantation using a full left lobe graft.

## METHODS

We report three cases of children weighing more than 20 kg who received complete left hemi-grafts in different clinical scenarios, all of which were successful. The indications and techniques adopted are discussed in the following sections.

## RESULTS

### Case reports

Case 1. An 11-year-old girl (58 kg) was diagnosed with fulminant liver failure due to autoimmune hepatitis. Meeting the King’s College criteria for liver transplantation^25^, she is listed with an operational PELD score of 30.2^4^. Possible candidates for a living donor are investigated, and the father meets the appropriate criteria, with a total hepatic volume of 1493.7 cc, left lobe 586.9 cc, and conventional vascular and biliary anatomy. The calculation of residual liver volume yields 60.7% for left hepatectomy, with a graft-to-body weight ratio (GBWR) of 1.01. On May 22, 2020, a liver transplant was performed with a living donor, involving a complete left hepatectomy in the donor with reconstruction and implantation of the middle and left hepatic veins into a common trunk to the recipient’s vena cava.

In the postoperative period, the recipient patient developed a biloma, which was percutaneously drained, and non-occlusive thrombosis of the middle suprahepatic vein was managed with anticoagulation with no evidence of thrombosis at 3 months. At 12 months post-transplant, she presented with late biliary stenosis, requiring reoperation with hepaticojejunostomy re-anastomosis, showing favorable outcomes without recurrence after more than 2 years of follow-up.

Case 2. A 7-year-old boy (20.2 kg) was diagnosed with hepatoblastoma with no metastases. He completed five cycles of neoadjuvant chemotherapy with an approximately 80% reduction in tumor mass volume. Considered unresectable and PRETEXT IV, he was listed for liver transplantation on July 31, 2020, with an operational PELD score of 30. Possible candidates for a living donor were investigated, and the mother met the appropriate criteria. The donor’s volumetry showed a total hepatic volume of 1066 cc, complete left lobe 367.2 cc, and left lateral lobe 235 cc. Regarding vascular anatomy, there was a cystic artery arising from the left hepatic artery, and concerning biliary anatomy, the left hepatic duct was 2 mm from the confluence.

The calculation of residual liver volume was 65.6% for left hepatectomy with a GBWR of 1.84. On August 5, 2020, a liver transplant was performed with a living donor, involving a complete left hepatectomy in the donor without the MHV, with anastomosis of the left hepatic vein to the recipient’s vena cava.

On the sixth postoperative day, the recipient required reoperation for bleeding and evacuation of hematoma. Following this, he showed favorable progress and was discharged to complete the chemotherapy regimen. He has not experienced other complications after more than 2 years of follow-up.

Case 3. A 13-year-old girl (31 kg) was diagnosed with chronic liver damage due to autoimmune hepatitis diagnosed at the age of 1 year. She developed portal hypertension, hepatopulmonary syndrome, Cushing’s syndrome, and short stature associated with the chronic use of corticosteroids. She was listed for liver transplantation on May 17, 2022, with an operational MELD score of 25. Possible candidates for a living donor were investigated, and the mother met the appropriate criteria. The donor’s volumetric assessment showed a total hepatic volume of 1247.9 cc, complete left lobe 426.7 cc, and left lateral lobe 264.3 cc. Vascular anatomy appeared conventional, and regarding biliary anatomy, the posterior right duct reaches the central left duct.

The calculation of residual liver volume yields 65.8% for left hepatectomy with a GBWR of 1.38. On August 3, 2022, a liver transplant was performed with a living donor, involving a complete left hepatectomy in the donor with anastomosis of the middle and left hepatic veins to the recipient’s vena cava. The left bile duct was sectioned distally to the confluence with the posterior right duct, preserving it. The recipient showed favorable progress and was discharged on the 17th postoperative day. At 6 months post-transplant, a liver biopsy was performed, revealing mild cellular rejection. Immunosuppression was adjusted. She has not experienced other complications after more than 1 year of follow-up.

## DISCUSSION

The low annual rate of deceased donors in Chile results in a high mortality rate on the waiting list for liver transplantation^9^, affecting both adult and pediatric recipients. Liver transplantation with a living donor stands out as a primary alternative to mitigate this high mortality on the waiting list^8,17^. Although the experience with adult-to-pediatric living donor liver transplantation in Chile has been substantial over the last 20 years, it has predominantly involved the use of left lateral segment grafts for recipients weighing less than 20 kg.

In 2022, 23 liver transplantations with living donors were performed in our country, with 19 conducted at our center^9,31^. This increased experience with living donors, in comparison to previous years, has helped narrow the gap between organ demand and supply, reducing waiting times and contributing to a decrease in mortality on the waiting list^6^. The experience from other centers, emphasizing this donation strategy, has shown excellent results, with some series reporting higher survival rates with living donors in children when adjusting for variables such as age, clinical characteristics, and time on the waiting list^6^. It is recognized that developing a program for adult and pediatric living donors entails significant technical and ethical challenges, necessitating ongoing collaboration from a multidisciplinary team and rigorous protocols for the monitoring of donors and recipients^12^. The focus remains on reducing donor morbidity without compromising outcomes for the recipient.

In terms of the surgical technique, four key elements have been identified as crucial for achieving a successful outcome in a liver transplant with a living donor:

1. Sufficient graft volume,
2. Appropriate irrigation or inflow,
3. Adequate venous drainage or outflow, and
4. Secure biliary anastomosis^20^.

All these factors interact collectively to determine the post-transplant graft function.

Regarding graft volume, traditionally, in adult-to-adult living donor liver transplantation, a hemi-graft has been used, either the right lobe or the complete left lobe, to ensure an adequate graft size^11,20^. However, since donor morbidity increases progressively with a larger hepatectomy volume, it is imperative to minimize the graft volume to the absolute necessity for the recipient, aiming to reduce donor complication rates^2,28^. In the case of pediatric recipients, the left lateral segment has been the preferred choice, providing an adequate volume for children weighing up to 20 kg and imposing a minor risk to the donor^18,29^.

In the selection of the donor liver segment, the risk of donor mortality has been described as up to 0.3% for an adult recipient and up to 0.1% for a pediatric recipient (left lateral segment). Regarding the type of hemi-graft, whether right or left lobe, no significant differences in mortality have been demonstrated. However, it is noted that the recovery time for hepatic function is shorter in the case of the right remnant, which aligns with the shorter hospitalization times reported for left lobe graft donors^16^. In relation to pediatric recipients, a non-concurrent cohort study conducted in the United States with approximately 3,000 patients concluded that survival is not affected by the type of implanted graft. This is consistent with a 2012 study that compiled experiences from transplant centers in North America, Europe, and Asia^5,16^.

Concerning surgical techniques to modulate inflow in cases of a small graft for the recipient (“small-for-size”), methods such as portocaval shunt, splenectomy, and ligation or embolization of the splenic artery are employed^15^. Similarly, outflow must be carefully evaluated, as hepatic congestion can lead to graft failure. These alterations are more frequently described in right grafts compared to left grafts, particularly if the MHV is not re-implanted. However, the decision to include the MHV in the right grafts is controversial, as preserving the MHV in the donor enhances the function and regeneration of the remnant^3,4,13,22,24^.

In the case of the left graft, various techniques have been described to ensure adequate outflow^15,20,23,30^. In pediatric recipients, when the graft size approaches the limit for the recipient’s size and weight, it may be necessary to include the MHV in the graft. This is achieved through reconstruction with an end-to-side anastomosis between the inferior vena cava (IVC) and a common trunk formed by the middle and left suprahepatic veins.

Graft volume, inflow, and outflow are directly related to the pathophysiology of the SFSS, a clinical condition that occurs when the graft is insufficient to meet the recipient’s demands. It is characterized by jaundice, ascites, coagulopathy, and encephalopathy. SFSS originates when the graft is unable to handle portal perfusion pressure, leading to portal hypertension, causing endothelial damage with increased permeability in sinusoids and bacterial translocation. Histologically, it is characterized by sinusoidal dilation and hepatocyte edema^7,14,27^. Classically, SFSS is described when the GBWR is less than 0.8%. However, with the advent of pharmacological and surgical techniques for portal flow reduction, successful cases of donation with a lower GBWR have been reported^19,21,26^.

This series of three cases of liver transplantation in larger children using a full left lobe graft, with adequate graft size, volume, and GBWR, and successful outcomes highlight the feasibility and safety of this technique as an alternative to the long deceased donor waitlist.

## CONCLUSIONS

The use of a living donor left lateral segment (segments 2 and 3) has been successfully employed in pediatric liver transplantation. However, it is only suitable for infants and low-weight children. This case series demonstrates that the techniques and experience gained from adult-to-adult living donor transplantation allow for the utilization of a complete left hemi-graft with or without MHV, as an alternative in pediatric transplantation for larger children. This approach contributes to the reduction of high mortality rates and waiting times associated with deceased donors.
